# Validation of an alternative technique for RQ estimation in anesthetized pigs

**DOI:** 10.1186/s40635-024-00598-8

**Published:** 2024-01-25

**Authors:** Jacob Karlsson, Anders Svedmyr, Mats Wallin, Magnus Hallbäck, Per-Arne Lönnqvist

**Affiliations:** 1 Department of Physiology and Pharmacology (FYFA), C3, PA Lönnqvist Group, Section of Anesthesiology and Intensive Care, Anestesi- och Intensivvårdsavdelningen, Karolinska Institute, 171 76 Stockholm, Sweden; 2 Pediatric Perioperative Medicine and Intensive Care, Karolinska University Hospital, Eugenivägen 23, 171 64 Stockholm, Sweden; 3 Maquet Critical Care AB, Röntgenvägen 2, 171 06 Solna, Sweden

**Keywords:** Respiratory quotient, Pigs, Calorimetry, Blood gas, Animal model

## Abstract

**Background:**

Respiratory quotient (RQ) is an important variable when assessing metabolic status in intensive care patients. However, analysis of RQ requires cumbersome technical equipment. The aim of the current study was to examine a simplified blood gas-based method of RQ assessment, using Douglas bag measurement of RQ (Douglas-RQ) as reference in a laboratory porcine model under metabolic steady state. In addition, we aimed at establishing reference values for RQ in the same population, thereby generating data to facilitate further research.

**Methods:**

RQ was measured in 11 mechanically ventilated pigs under metabolic steady state using Douglas-RQ and CO-oximetry blood gas analysis of pulmonary artery and systemic carbon dioxide and oxygen content. The CO-oximetry data were used to calculate RQ (blood gas RQ). Paired recordings with both methods were made once in the morning and once in the afternoon and values obtained were analyzed for potential significant differences.

**Results:**

The average Douglas-RQ, for all data points over the whole day, was 0.97 (95%CI 0.95–0.99). The corresponding blood gas RQ was 0.95 (95%CI 0.87–1.02). There was no statistically significant difference in RQ values obtained using Douglas-RQ or blood gas RQ for all data over the whole day (*P* = 0.43). Bias was − 0.02 (95% limits of agreement ± 0.3). Douglas-RQ decreased during the day 1.00 (95%CI 0.97–1.03) vs 0.95 (95%CI 0.92–0.98) *P* < 0.001, whereas the decrease was not significant for blood gas RQ 1.02 (95%CI 0.89–1.16 vs 0.87 (0.80–0.94) *P* = 0.11.

**Conclusion:**

RQ values obtained with blood gas analysis did not differ statistically, compared to gold standard Douglas bag RQ measurement, showing low bias but relatively large limits of agreement, when analyzed for the whole day. This indicates that a simplified blood gas-based method for RQ estimations may be used as an alternative to gold standard expired gas analysis on a group level, even if individual values may differ. In addition, RQ estimated with Douglas bag analysis of exhaled air, was 0.97 in anesthetized non-fasted pigs and decreased during prolonged anesthesia.

**Supplementary Information:**

The online version contains supplementary material available at 10.1186/s40635-024-00598-8.

## Background

The porcine model is frequently used in laboratory research, e.g., in cardiorespiratory, metabolic and sepsis studies. To investigate some of these specific research questions will require either actual measurement or adequate published data regarding the respiratory quotient (RQ). RQ is for instance frequently used for estimation of oxygen consumption and in metabolic animal studies. Since data used from tabulated RQ values may lead to inaccurate calculations, it would therefore be desirable to establish alternative methods to traditional RQ measurement techniques. When further developing a novel method for non-invasive and continuous determination of cardiac output (CO), we realized the need for determination of the RQ in anesthetized pigs to calculate non-invasive mixed venous oxygen saturation (SvO_2_) [1]. Traditionally, RQ has been measured by relatively cumbersome methods such as Douglas bag or indirect calorimetry, which are not always available [2]. In addition, these methods are associated with inherent uncertainties related to the requirements of high precision gas analysis. We therefore wanted to examine if RQ can be adequately estimated during metabolic steady state via a simplified technique based on blood gas analysis of pulmonary arterial oxygen and carbon dioxide (CO_2_) content in combination with systemic arterial oxygen and CO_2_ content, using Douglas bag as comparison method.

Thus, the aim of our current study was to examine this simplified blood gas-based method of RQ assessment using Douglas bag measurement of RQ as reference in a laboratory porcine model under metabolic steady state, while using an established animal anesthesia protocol [1].

In addition, we aimed at establishing reference values for RQ in the same population, using gas analysis of Douglas bag collected exhaled air, thereby generating data to facilitate further research.

## Methods

After the approval of the Uppsala Animal Ethics Committee (case number C75/16, chairperson Erik Göransson on August 26, 2016), the study was conducted at the Hedenstierna laboratory in Uppsala, Sweden involving 11 domestic-breed pigs of both sexes (Sus scrofa domesticus). The animals were treated in accordance with the animal experimentation guidelines established by the Uppsala Animal Ethics Committee and the Animal Research: Reporting of In Vivo Experiments (ARRIVE) guidelines [3].

### Anesthesia protocol

A total of 11 domestic-breed pigs of both sexes, median weight 29.6 kg (range 27.1–31.7 kg) aged between 6 and 8 weeks, were utilized in the study. All pigs were sourced from the same breeding colony (Mångsbo Farm, Uppsala, Sweden), and were kept in a controlled environment with regulated temperature and lighting. The animals were provided with unrestricted access to tap water and food, provided on a consistent schedule up till one hour prior to anesthesia induction. All experiments were conducted during daylight hours. The animals in the current study were also included in another analysis, assessing the performance of a novel SvO_2_ estimation method [1].

An intramuscular dose of atropine (0.04 mg kg^−1^, NM Pharma AB, Sweden), tiletamine–zolazepam (6 mg kg^−1^, Zoletil, Virbac Laboratories, France), and xylazine chloride (2.2 mg kg^−1^, Rompun, Bayer AG, Germany) was administered for induction of anesthesia. For anesthesia maintenance, a combination of fentanyl bolus (5 μg kg^−1^, Fentanyl B. Braun, Germany), ketamine (30 mg kg^−1^ h^−1^), midazolam (0.1 mg kg^−1^ h^−1^), and fentanyl infusion (4 μg kg^−1^ h^−1^) was administered in combination with muscle relaxation using rocuronium (bolus 1 mg kg^−1^ followed by infusion 2 mg kg^−1^ h^−1^). After adequate level of anesthesia was ensured (see below), the airway was secured using a surgical tracheostomy with a standard cuffed endotracheal tube. Laboratory standard monitoring procedures were employed throughout the experiment to maintain appropriate levels of anesthesia and analgesia. This includes continuous assessment of sympathetic reactions as demonstrated by hemodynamic variations, i.e., variations in heart rate and blood pressure and reaction to eyelash brush well as motor response following sympathetic stimulus.

In addition to the standard monitoring, a 7.5F pulmonary artery catheter (Swan-Ganz pulmonary artery catheter, model 774F75; Edwards Lifesciences, Irvine CA; USA) and a 5F femoral artery cannula were inserted for blood gas sampling and temperature measurement (target temperature 38 C). Temperature was regulated using heated mattress and temperature regulation of the intravenous maintenance fluids. The animals were also fitted with additional monitoring devices intended for further cardiac output studies, which have been described in detail in previous studies [1]. Blood gases were analyzed via blood samples using a CO-oximeter calibrated specifically for porcine hemoglobin (OSM3; Radiometer Medical AbS, Brønshøj, Denmark), which also allowed for the determination of pulmonary mixed venous and arterial CO_2_ and O_2_ content required for blood gas-based RQ calculations. After the experiment, the animals were euthanized using intravenous potassium chloride (KCl: 100 mmol) via the central venous catheter as per established standard at the laboratory.

### Ventilation and maintenance fluids

The pigs were placed under mechanical ventilation using a volume-controlled mode with a tidal volume of 10 ml kg^−1^, fraction of inspired oxygen (FiO_2_) of 0.3. Positive end-expiratory pressure (PEEP) was maintained at 5 cm H_2_O after an initial 2-min lung recruitment using PEEP of 10 cm H_2_O with constant driving pressures as described previously [4]. An air test was then performed using FiO_2_ of 0.21 to assess sustained pulse oximetry saturation > 97% [5]. The lung recruitment was repeated if necessary to ensure open lung conditions. Expiratory CO_2_ levels were measured using a mainstream infrared CO_2_ sensor (Capnostat-3; Respironics Inc, Wallingford, CT), and ventilation airflow was monitored through the regular flow sensors of the Servo-I ventilator (Maquet, Solna, Sweden).

The pigs were administered a bolus of Ringers’ acetate solution of 20 ml kg^−1^ after induction and then kept on a maintenance infusion of glucose 25 mg ml^−1^ at a rate of 8 ml kg^−1^ h^−1^ and Ringer’s acetate solution at a rate of 10 ml kg^−1^ h^−1^.

### Determination of RQ using Douglas bag

RQ was measured once in the morning and once in the afternoon, with approximately a 4-h interval between measurements. The RQ was measured in all animals by collecting mixed expired gas in a Douglas bag.

### Derivation of the RQ-equation

The use of Douglas bag gas collection and analysis allows for the determination of the respiratory exchange ratio (RER). This measures the ratio of carbon dioxide elimination (VCO_2_) to oxygen uptake (VO_2_). When in a state of equilibrium, RER should match the respiratory quotient (RQ). RER is related to the uptake (VO_2_) and elimination (VCO_2_) of oxygen and carbon dioxide as described in Eq. 1:

1 $${\text{RQ}}={\text{RER}}=\frac{{{\text{VCO}}}_{2}}{{{\text{VO}}}_{2}}.$$

VCO_2_ and VO_2_ are determined by inspired and expired tidal volumes and mixed expired volume fractions of the gases (assuming that inspired gas is free from CO_2_):

2 $${{\text{VCO}}}_{2}={\text{Ve}}\cdot {{\text{FemixCO}}}_{2},$$

3 $${{\text{VO}}}_{2}={\text{Vi}}\cdot {{\text{FiO}}}_{2}-{\text{Ve}}\cdot {{\text{FemixO}}}_{2}.$$

The relation between inspired and expired volumes needs to be very precisely determined and to this end nitrogen (N_2_, here also implicitly including argon) can be used as an inert balance gas with zero net exchange:

4 $${{\text{VN}}}_{2}={\text{Vi}}\cdot {{\text{FiN}}}_{2}-{\text{Ve}}\cdot {{\text{FemixN}}}_{2}=0.$$

Thus, based on this strategy (often referred to as the Haldane transformation) inspired volume can be related to expired volume and the balance gas concentrations:

5 $${\text{Vi}}={\text{Ve}}\cdot \frac{{{\text{FemixN}}}_{2}}{{{\text{FiN}}}_{2}}.$$

The nitrogen fractions are given by

6 $${{\text{FiN}}}_{2}=1-{{\text{FiO}}}_{2},$$

7 $${{\text{FemixN}}}_{2}=1-{{\text{FemixO}}}_{2}-{{\text{FemixCO}}}_{2}.$$

Substitution of all these relations into the RER and RQ expression as shown in Eq. 1 above, yields after some algebraic simplifications (complete algebraic simplification shown in Additional file 1) Eq. 8:

8 $${\text{RQ}}=\frac{\left(1-{{\text{FiO}}}_{2}\right)\cdot {{\text{FemixCO}}}_{2}}{\left(1-{{\text{FemixCO}}}_{2}\right)\cdot {{\text{FiO}}}_{2}-{{\text{FemixO}}}_{2}}.$$

During the experiment, the gas collected in the Douglas bag contains water vapor that is exhaled, which is not accounted for in the equations above. This water vapor increases the volume of the gas and reduces the concentration of other gas components. However, if the water vapor is eliminated, then the equations that were developed for dry gases would be valid. This is achieved by sampling and drying the gas before it enters the analysis chamber of the side-stream gas analyzer system. If the gas concentrations are reported in a dry state (ATPD, Ambient temperature and pressure, dry), then Eq. 8 can be used to determine the respiratory quotient (RQ).

The procedure for each RQ measurement was as follows:

- To avoid dilution of the gas in the bag with inspiration gas, the ventilator bias flow during the expiration phase was turned off.
- FiO_2_ was decreased from 0.30 to 0.21 (i.e., room air). This was done to optimize the conditions for RQ calculation (as shown in Eq. 1), as well as ensuring a well-defined inspired gas concentration without depending on accurate gas mixing by the ventilator.
- To establish a steady-state of nitrogen (N_2_), a 20-min wash-out of O_2_ was conducted after the change of FiO_2_ and prior to connecting the bag to the ventilator exhaust port.
- Gas was collected for approximately 10 min, during which roughly 70 L of expiratory gas were collected.
- The content of the bag was analyzed using the side-stream gas analyzer of a Flow-i anesthesia system (Maquet, Solna, Sweden fitted with AION gas analyzer). Gas was sampled alternately from the bag and room air.

Gas concentrations of FiO_2_ (room air), FemixO_2_ (bag) and FemixCO_2_ (bag) were measured at ATPD state (Ambient temperature and pressure, dry).

### Blood gas-based RQ estimation

Paired pulmonary arterial mixed venous and systemic arterial blood gases were used to determine blood gas RQ. Since RER should equal RQ at steady state (i.e., VCO_2_/VO_2_) as shown in Eq. 1 above, the difference in CO_2_ content between pulmonary arterial mixed venous blood (C_v_CO_2_) and systemic arterial blood (C_a_CO_2_) can be divided by the corresponding difference for O_2_ content (i.e., C_a_O_2_ and C_v_O_2_) to yield RQ as shown in Eq. 9 below:

9 $${\text{RQ}} = \frac{{{\text{C}}_{{\text{v}}} {\text{CO}}_{2} - {\text{C}}_{{\text{a}}} {\text{CO}}_{2} }}{{{\text{C}}_{{\text{a}}} {\text{O}}_{2} - {\text{C}}_{{\text{v}}} {\text{O}}_{2} }}$$

Equation 9 is obtained by cancelling out CO since CO = VCO_2_/(CvCO_2_–CaCO_2_) which is also true for oxygen as per Fick’s principle, i.e., CO = VO_2_/(CaO_2_–CvO_2_). VCO_2_/VO_2_ (i.e., RQ) thus equals (CO^.^ (CvCO_2_–CaCO_2_))/(CO^.^ (CaO_2_–CvO_2_)) where CO can be mathematically cancelled out. Mixed venous and arterial CO_2_ content were calculated by the incorporated algorithm in the blood gas machine which calculates the sum of total CO_2_ in plasma (P) and erythrocyte fluid (Ery) as previously described in detail [6, 7]. Mixed venous and arterial O_2_ content were calculated as the sum of free and bound oxygen as previously described in detail [7]. Both CO_2_ and O_2_ content were thus calculated automatically by the arterial blood gas machine calibrated for porcine hemoglobin. A detailed description of the calculation of CO_2_ and O_2_ content, as presented in reference 7, is shown in Additional file 1.

Mixed venous and arterial blood gases for blood gas RQ analysis were sampled under steady-state conditions just before the Douglas-based RQ estimations. All blood samples were analyzed by a CO-oximeter calibrated for porcine hemoglobin (OSM3; Radiometer Medical AbS, Brønshøj, Denmark) as well as with a standard blood gas analyzer for additional parameters (ABL800FLEX, Radiometer Medical AbS, Brønshøj Denmark).

Glucose levels were measured from arterial blood gases in the morning and afternoon to compare any changes between measurements.

### Statistical analysis

The data for RQ for both methods were checked for normal distribution using the D'Agostino and Pearson test, as well as visual inspection of the corresponding histograms. The values are reported as the mean and 95% confidence interval (CI).

Difference between Douglas-RQ and blood gas RQ for the morning and afternoon measurements as well as for the whole day, was analyzed using Wilcoxon matched-pairs signed rank test. Variations in RQ between morning and afternoon for both Douglas-RQ and blood gas RQ and for blood glucose were analyzed for potential significant change, using Wilcoxon matched-pairs signed rank test. A value of *P* < 0.05 was consider indicative for statistical significance. Agreement of absolute values between Douglas-RQ and blood gas RQ was assessed using Bland–Altman analysis with bias (i.e., mean difference between the tested methods and the reference method) and the corresponding 95% limits of agreement. The ability of blood gas RQ to detect change was assessed using concordance analysis. An exclusion zone of 5% was used based on previous publication on inherent precision for metabolic monitors [8, 9]*.*

GraphPad Prism (version 9.0 for Windows, GraphPad Software, San Diego, CA, USA) was used for statistical calculations and Microsoft Excel for Mac 2021 version 16.45 for data handling.

## Results

Individual data for each subject in the cohort are displayed in Table 1 together with the RQ data with reference values for awake starved and non-starved pigs [10, 11].

**Table 1:** Individual RQ values obtained with both reference method (RQ Douglas) and tested method (RQ blood gas)

	RQ Douglas	RQ blood gas	RQ Douglas	RQ blood gas	RQ Douglas	RQ blood gas
	Mean AM + PM	Mean AM + PM	AM	AM	PM	PM
1	0.92	1.03	0.96	1.35	0.88	0.7
2	0.92	0.99	0.96	1.02	0.89	0.96
3	1.02	0.91	1.04	0.87	1	0.96
4	0.98	1.04	1.01	1.19	0.96	0.89
5	0.99	1.02	1.02	1.19	0.95	0.85
6	0.94	1.06	0.96	1.17	0.91	0.96
7	1.03	0.88	1.06	0.83	1	0.94
8	0.96	0.87	0.95	0.84	0.98	0.91
9	0.96	1.03	1.01	*	0.91	1.03
10	0.98	0.86	1.03	0.91	0.94	0.82
11	1.01	0.77	1.02	0.82	1	0.72
Mean (95%CI)	0.97 (0.95–0.99)	0.95 (0.87–1.02)	1.00 (0.97–1.03)	1.02 (0.89–1.16)	0.95 (0.92–0.98)	0.87 (0.80–0.94)
RQ awake pig starved 24 h	0.87					
RQ awake pig non-starved	1.07					

*Missing data subject 9. Values are mean 95%CI

The average Douglas-RQ, for all data points over the whole day, was 0.97 (95%CI 0.95–0.99). The corresponding value using blood gas RQ was 0.95 (95%CI 0.87–1.02). There was no statistically significant difference in RQ values obtained using Douglas-RQ or blood gas RQ for all data over the whole day (*P* = 0.43). Bland Altman analysis showed a bias of -0.023 with 95% limits of agreement ± 0.3. Eight data points were included in the concordance analysis, of which one blood gas RQ value did not change in the same direction as the reference method, corresponding to a concordance rate of 88%.

A small but statistically significant difference in RQ was noted between the morning and the afternoon value for Douglas-RQ, 1.00 (95%CI 0.97–1.03) vs 0.95 (95%CI 0.92–0.98) *P* = 0.0039 but not for blood gas RQ 1.02 (95%CI 0.89–1.16 vs 0.87 (0.80–0.94) *P* = 0.11.

When comparing morning values between Douglas-RQ and blood gas RQ, no significant difference was found *P* = 0.92. A small but significant difference was seen when comparing the afternoon values for Douglas-RQ and blood gas RQ (*P* = 0.049).

**Fig. 1:**
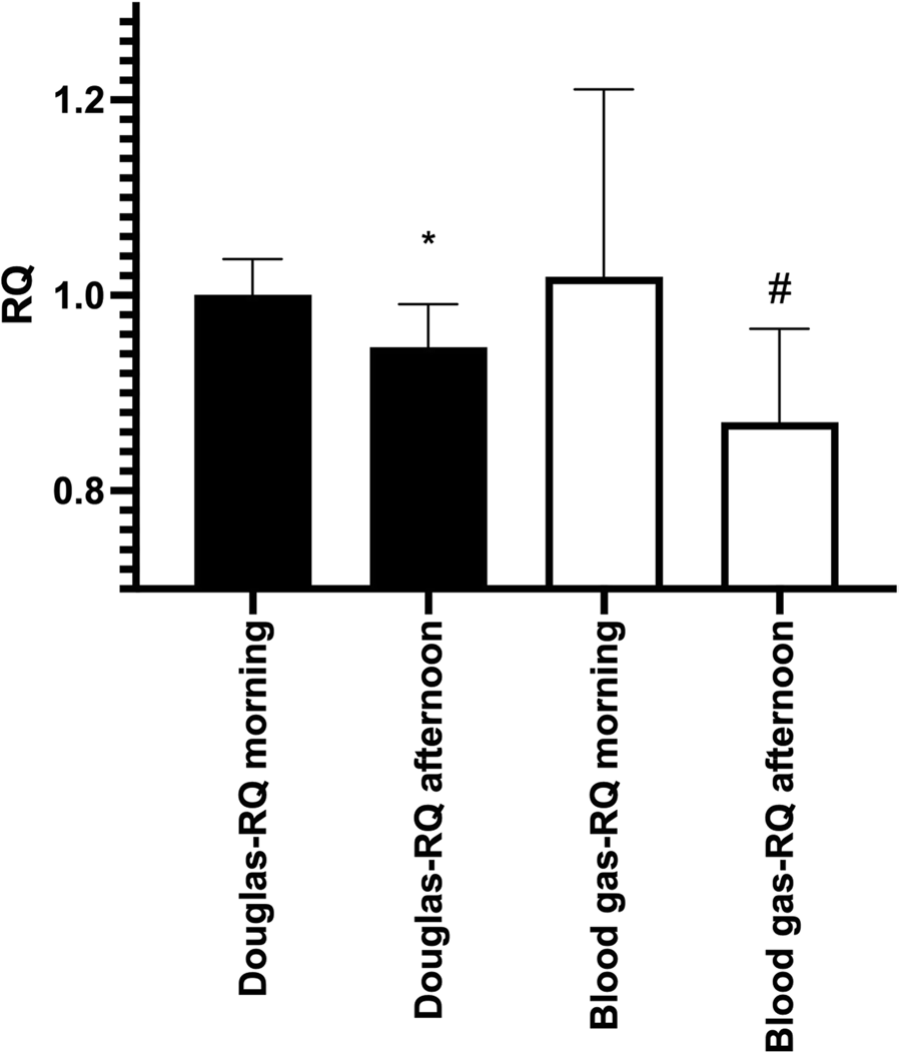
RQ values obtained using Douglas bag and blood gas. *P < 0.05 compared to morning values. ^#^P < 0.05 Douglas vs blood gas. Values are mean (95%CI). *N* = 10–11

Mean RQ values for Douglas and blood gas-based methods in the morning and afternoon are shown in Fig. 1.

**Fig. 2:**
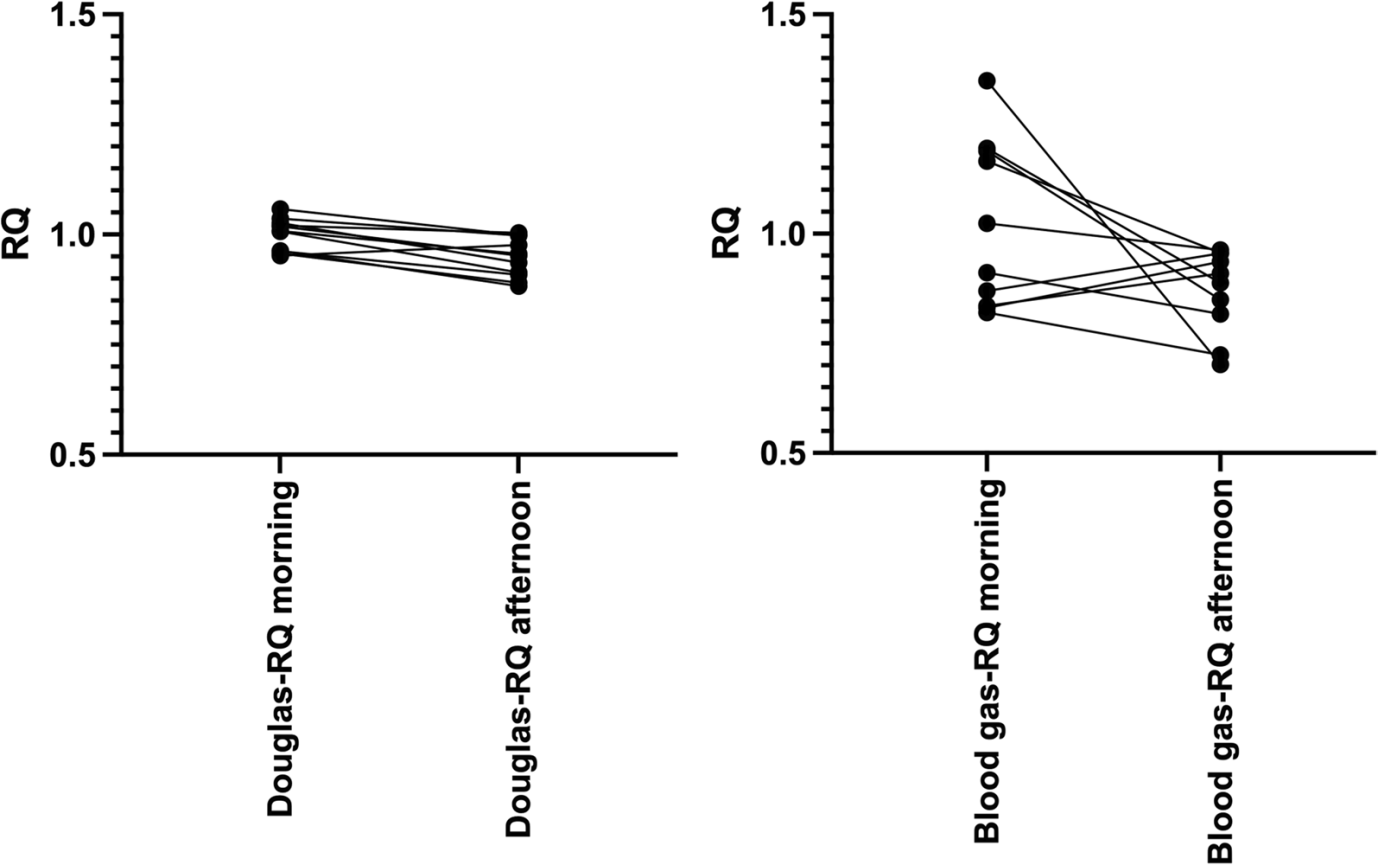
The individual changes in RQ for both methods during the day. *N* = 10–11

The individual change in RQ from Table 1 is graphically displayed in Fig. 2 to illustrate the change in RQ over the course of the day.

Blood-glucose decreased significantly from 9.3 mmol/L (95%C 8.0–10.7 mmol/L) to 5.5 mmol/L (5.1–5.9 mmol/L) *P* = 0.0020 when comparing morning and afternoon samples.

## Discussion

The main finding of the present study was that values obtained using Douglas-RQ did not differ significantly from values obtained using the simplified blood gas-based method when analyzed for the whole day. The agreement of absolute values assessed as bias between blood gas RQ and Douglas RQ was acceptable, albeit with relatively wide limits of agreement. This suggests that blood gas-based analysis of RQ, may serve as an alternative to the more cumbersome expired gas analysis of RQ in the laboratory setting on group level, even if individual values may still differ, thus potentially limiting its usefulness on individual level.

In addition, the overall mean value of RQ during steady-state anesthesia in pigs was found to be 0.97 (95%CI 0.95–0.99) assessed using mixed expired gas analysis. Furthermore, the RQ value was found to decrease during prolonged anesthesia.

Since tabulated values of RQ in pigs appears to be potentially misleading, a readily available and accurate RQ estimation method such as blood gas-based RQ may therefore result in more accurate calculations. If for example an RQ of 0.8 is used to calculate a VO_2_ value from a known VCO_2_ value, this would lead to an approximately 20% overestimation of VO_2_ (0.97/0.8 = 1.21, i.e., 21%) which may further affect for instance cardiac output calculations based on classic Fick equation [12, 13].

Metabolic, cardiorespiratory and sepsis studies performed in the laboratory environment may require adequate knowledge of the RQ since this value varies in-between species (typical values for anesthetized humans is approximately 0.85). Since anesthesia substantially reduces the metabolic requirements of the intended research subject compared with the awake state [14], it is in certain situations both desirable and important to have adequate knowledge regarding the RQ in the specific setting. However, as alluded to the introduction, in a previous proof-of-concept study regarding non-invasive determination of SvO_2_, which required knowledge of RQ, we could only find RQ values for awake pigs in the fed or starved state [10, 11]. In fact, a relatively large body of work regarding RQ in awake pigs exists, mainly from agricultural research. To use such RQ values from the awake state with various nutritional status is obviously suboptimal in the context of anesthesia in a research setting. Thus, the finding that RQ appears to be substantially different between reference values for awake state and the values in an anesthetized laboratory state, may be of help for research groups performing scientific studies in anesthetized pigs where RQ is used.

### Awake vs. anesthetized

As can be seen in Table 1 the RQ values in anesthetized pigs is about 10% less during anesthesia than in the fed awake state, and 10% higher as compared to the awake starved state, thus indicating the nutritional substrates importance for the balance between VO_2_ and VCO_2_ in addition to sedation state [10, 11]. This is in line with data in humans where RQ is typically approximately 0.85 in awake fed individuals and 0.7 in awake starving subjects [15]. The corresponding value in anesthetized humans also demonstrates that the choice of substrate affects RQ in a similar way (i.e., anesthetized starved; 0.81 and non-starved: 0.92 [16]. Anesthesia thus appears to influence RQ to a lesser extent than degree of starvation and choice of nutritional substrate in both humans and pigs.

### Effects of prolonged anesthesia

Overall, the RQ was observed to be slightly reduced after a prolonged anesthetic, as compared to the value measured immediately following induction of anesthesia (Table 1 and Fig. 1). This effect was also accompanied by a significant reduction in blood glucose levels potentially indicating a less carbohydrate-based metabolism towards the end of the day which may explain the decrease seen in RQ. It is however doubtful whether this has any implications in the general research setting or have any translational implications. Against this background it appears reasonable to use the study all-day average value in situations where the RQ does not need to be individually assessed due to strict research demands.

### Effect of substrate

The animals in the current study were given a glucose maintenance infusion of 25 mg ml^−1^ 8 ml kg^−1^ h^−1^ corresponding to approximately 3 mg glucose kg^−1^ min^−1^. This pure carbohydrate substrate results in a very different VCO_2_ production compared to more fat and protein concentrated nutrition [17]. This will affect the estimated RQ values, and it is therefore important to keep in mind that the RQ values obtained in the current study should only be used for anesthetized pigs with carbohydrate maintenance of the same magnitude. This nutritional strategy represents, however, a standard laboratory nutrition and should therefore be possible to integrate in further calculations related to similar animal research porcine models [18].

### Limitations

Firstly, the current study used piglets with high metabolic turn over. This may not reflect the situation in older pigs where the CO_2_ production may be lower. Available data from piglets and adult pigs have however demonstrated relatively small differences between piglets and larger pigs (e.g., approximately 0.97 for fed piglets, compared to 1.0 for awake fed sows [19, 20]. It is therefore possible that the RQ values obtained in the current study may also be applied in models using larger pigs even if this was not investigated.

In this context, it is also important to emphasize that all measurements were made under metabolically stable conditions and the performance of blood gas RQ and Douglas derived RQ in scenarios with major changes in metabolic turn over were not investigated in the current study (e.g., challenges with insulin and epinephrine infusions).

Secondly, even if the absolute values of RQ statistically were not different between both measurement methods when analyzed for the whole day, the spread of the data points were larger when blood gas RQ was used. This should be kept in mind when assessing individual RQ values and may be attributed to the precision of the blood gas analysis, particularly when analyzing blood gases from mixed venous blood where inherent precision has been shown to be higher than on the arterial side [21, 22]. This is illustrated in the larger 95%CI seen for the morning and afternoon values for blood gas RQ.

This may also have contributed to the small but significant difference seen when comparing the afternoon values for Douglas-RQ and blood gas RQ (*P* = 0.049).

Thirdly, the calculations of systemic arterial and mixed venous CO_2_ content may also be affected by factors shifting the CO_2_ dissociation curve. Importantly, hypoxia alters the CO_2_ content in whole blood by increasing CO_2_ loading and shifting the CO_2_ dissociation curve upwards via the reverse Haldane effect and this scenario was not investigated in the current study. Additional factors such as acidosis and alterations in oxygen dissociation curve, may also affect calculated O_2_ content and this was not investigated in the current study.

It is important to emphasize that also the reference method, Douglas bag measurement of RQ, is associated with uncertainties (mainly related to the requirement of high precision gas analysis) and just like the blood gas method this must be considered when assessing individual RQ values, even if each method appears suitable for estimating RQ in a population. In addition, precision measurements are not practically possible to perform with Douglas bag or alternative gas mixing chamber methods and thus their inherent precision and accuracy on individual level is associated with uncertainties**.** Previous studies have also shown a relatively large variation between different types of gas mixing chambers thus further illustrating the potential uncertainty associated with these methods [23]. It is therefore important to remember that even if Douglas bag in this case was used as reference, in practice the performance of all gas mixing chambers as “gold standard” is still dependent on the accuracy and inherent precision of the gas analysis and ability to deliver stable fraction of inspired oxygen. We have illustrated this in a calculation example in Additional file 1.

The RQ estimation made with Douglas bag in the current study was made under as accurate and stable conditions as possible, unlikely to be encountered in for instance clinical practice. Since different metabolic monitors have shown a large discrepancy in agreement also when compared to Douglas bag, it can also be added as a limitation that no additional “gold standard” RQ monitoring method was used for comparison between Douglas bag, blood gas RQ and alternative metabolic monitoring (e.g., Deltatrac^®^ (Datex-Ohmeda, Helsinki, Finland).

### Potential clinical application

In addition to providing normal values for RQ in anesthetized pigs, the current study also represents a validation of simplified blood gas-based approach for RQ estimation. In an intensive care environment, assessment of RQ may be desired for metabolic monitoring as well as nutritional guidance [24]. This is normally done by using a dedicated metabolic monitor which requires specialized equipment and training [23]. If RQ instead can be reasonably accurately estimated in a stable subject via blood gas analysis, this would likely represent a simplification of currently available methods, albeit that the approach requires pulmonary artery catheter in addition to systemic arterial blood sampling.

## Conclusions

RQ values obtained with Douglas bag did not differ statistically from RQ calculations based on mixed venous and systemic arterial blood gas analysis when analyzed for the whole day. Agreement of absolute values was good but associated relatively large limits of agreement. This indicates that a simplified blood gas-based method for RQ estimations may be further explored as an alternative to expired gas analysis. In addition, RQ estimated with Douglas bag analysis of exhaled air, was 0.97 in anesthetized non-fasted pigs and decreased during prolonged anesthesia.

### Supplementary Information

**Additional file 1.**  Appendix 1.

## Data Availability

The datasets used and/or analyzed during the current study are available from the corresponding author on reasonable request.

## Abbreviations

ATPD: Ambient temperature and pressure, dry
CaCO_2_: CO_2_ content systemic arterial blood
CaO_2_: O_2_ content systemic arterial blood
CI: Confidence interval
CO_2_: Carbon dioxide
CvCO_2_: CO_2_ content pulmonary arterial blood
CvO_2_: O_2_ content pulmonary arterial blood
Femix O_2_: Mixed expired volume fraction O_2_
FemixCO_2_: Mixed expired volume fraction CO_2_
FemixN_2_: Mixed expired volume fraction N_2_
FiN_2_: Inspired fraction N_2_
FiO_2_: Inspired fraction O_2_
N_2_: Nitrogen gas
O_2_: Oxygen gas
PEEP: Positive end-expiratory pressure
RER: Respiratory exchange ratio
RQ: Respiratory quotient
SvO_2_: Mixed venous oxygen saturation
VCO_2_: Volumes of exhaled CO_2_
Ve: Expired volume
Vi: Inspired volume
VN_2_: Volume nitrogen, difference between inspired and expired
VO_2_: Volumes of exhaled O_2_
