# Supplementary material for: Validation of an alternative technique for RQ estimation in anesthetized pigs
Source: Intensive Care Med Exp. 2024 Jan 25;12:11. doi: 10.1186/s40635-024-00598-8 (PMC10811304; doi:10.1186/s40635-024-00598-8)
Supplement: Supplementary file 1 — Additional file 1. Appendix 1. [file 40635_2024_598_MOESM1_ESM.docx]

Appendix 1

| Douglas bag determination of RQ, an example of the influence of measurement sensitivity. | | | | | | | | | | | |  | |  | |  | |  | |  | |  | |  | |  | |  | |  | |  | |  | |  |  |
| --- | --- | --- | --- | --- | --- | --- | --- | --- | --- | --- | --- | --- | --- | --- | --- | --- | --- | --- | --- | --- | --- | --- | --- | --- | --- | --- | --- | --- | --- | --- | --- | --- | --- | --- | --- | --- | --- |
|  |  | |  | |  | | |  |  | | |  | |  | |  | |  | |  | |  | |  | |  | |  | |  | |  | |  | |  |  |
| Formula: |  | | | | | | | | | | | | |  | |  | |  | |  | |  | |  | |  | |  | |  | |  | |  | |  |  |
|  |  |  |  |  |  |  |  |  |  |  |  |  |  |  | |  | |  | |  | |  | |  | |  | |  | |  | |  | |  | |  |  |
|  |  |  |  |  |  |  |  |  |  |  |  |  |  |  | |  | |  | |  | |  | |  | |  | |  | |  | |  | |  | |  |  |
|  |  |  |  |  |  |  |  |  |  |  |  |  |  |  | |  | |  | |  | |  | |  | |  | |  | |  | |  | |  | |  |  |
|  |  | |  | |  | | |  |  | | |  | |  | |  | |  | |  | |  | |  | |  | |  | |  | |  | |  | |  |  |
| Case 1 is a baseline case, cases 2-4 are small deviations in each of the gas fraction values in red text and the associated effect on the RQ determination (deltaRQ, i.e., the change in RQ in response to deviations in gas fraction values). | | | | | | | | | | | | | |  | |  | |  | |  | |  | |  | |  | |  | |  | |  | |  | |  |  |
|  |  | |  | |  | | |  |  | | |  | |  | |  | |  | |  | |  | |  | |  | |  | |  | |  | |  | |  |  |
| **Case** | **FiO2** | | **FemixO2** | | **FemixCO2** | | | **RQ** | **deltaRQ** | | |  | |  | | | | | | | | | | | | | | | |  | |  | |  | |  |  |
| 1 | 0,210 | | 0,178 | | 0,030 | | | 0,922 | 0,000 | | |  | |  |  |  |  |  |  |  |  |  |  |  |  |  |  |  |  |  | |  | |  | |  |  |
| 2 | 0,211 | | 0,178 | | 0,030 | | | 0,888 | -0,035 | | |  | |  |  |  |  |  |  |  |  |  |  |  |  |  |  |  |  |  | |  | |  | |  |  |
| 3 | 0,210 | | 0,179 | | 0,030 | | | 0,960 | 0,037 | | |  | |  |  |  |  |  |  |  |  |  |  |  |  |  |  |  |  |  | |  | |  | |  |  |
| 4 | 0,210 | | 0,178 | | 0,031 | | | 0,961 | 0,039 | | |  | |  |  |  |  |  |  |  |  |  |  |  |  |  |  |  |  |  | |  | |  | |  |  |
|  |  | |  | |  | | |  |  | | |  | |  |  |  |  |  |  |  |  |  |  |  |  |  |  |  |  |  | |  | |  | |  |  |
| If FiO2 is measured too high, then RQ calculation will be too low. | | | | | | | | |  | | |  | |  |  |  |  |  |  |  |  |  |  |  |  |  |  |  |  |  | |  | |  | |  |  |
| If FemixO2 is measured too high, then RQ calculation will be too high. | | | | | | | | | | | |  | |  |  |  |  |  |  |  |  |  |  |  |  |  |  |  |  |  | |  | |  | |  |  |
| If FemixCO2 is measured too high, then RQ calculation will be too high. | | | | | | | | | | | |  | |  |  |  |  |  |  |  |  |  |  |  |  |  |  |  |  |  | |  | |  | |  |  |
|  |  | |  | |  | | |  |  | | |  | |  |  |  |  |  |  |  |  |  |  |  |  |  |  |  |  |  | |  | |  | |  |  |
| 0.1% unit error in gas concentration gives almost 0.04 units error in RQ, | | | | | | | | | | | |  | |  |  |  |  |  |  |  |  |  |  |  |  |  |  |  |  |  | |  | |  | |  |  |
| if for example FemixCO2 = 3.1% instead of 3.0%.  The Figure below illustrates the impact of the measurement errors listed above. | | | | | | | |  |  | | |  | |  |  |  |  |  |  |  |  |  |  |  |  |  |  |  |  |  | |  | |  | |  |  |
|  |  | |  | |  | | |  |  | | |  | |  |  |  |  |  |  |  |  |  |  |  |  |  |  |  |  |  | |  | |  | |  |  |
|  | |  | |  | |  |  | | |  | | | | | | | | | | | | | | |  | |  | |  | |  | |  | |  |  |  |
|  | |  | |  | |  |  | | |  |  |  |  |  |  |  |  |  |  |  |  |  |  |  |  | |  | |  | |  | |  | |  |  |  |
|  | |  | |  | |  |  | | |  |  | |  | |  | |  | |  | |  | |  | |  | |  | |  | |  | |  | |  |  |  |
|  | |  | |  | |  |  | | |  |  | |  | |  | |  | |  | |  | |  | |  | |  | |  | |  | |  | |  |  |  |
|  | |  | |  | |  |  | | |  |  | |  | |  | |  | |  | |  | |  | |  | |  | |  | |  | |  | |  |  |  |

Derivation of final equation 8 for RQ from equations 1-7.

Equation 1 $RQ=RER=\frac{VCO_{2}}{VO_{2}}$

VCO_2_ and VO_2_ are determined by inspired and expired tidal volumes and mixed expired volume fractions of the gases (assuming that inspired gas is free from CO_2_):

Equation 2 $VCO_{2}=Ve\cdot FemixCO_{2}$

Equation 3 $VO_{2}=Vi\cdot FiO_{2}-Ve\cdot FemixO_{2}$

The relation between inspired and expired volumes needs to be very precisely determined and to this end nitrogen (N_2_, here also implicitly including Argon) can be used as an inert balance gas with zero net exchange:

Equation 4 $VN_{2}=Vi\cdot FiN_{2}-Ve\cdot FemixN_{2}=0$

Thus, based on this strategy (often referred to as the Haldane transformation) inspired volume can be related to expired volume and the balance gas concentrations:

Equation 5 $Vi=Ve\cdot\frac{FemixN_{2}}{FiN_{2}}$

The nitrogen fractions are given by

Equation 6 $FiN_{2}=1-FiO_{2}$

Equation 7 $FemixN_{2}=1-FemixO_{2}-FemixCO_{2}$

Step by step calculation that leads to equation 8:

Combine Equation 1 with Equations 2 and 3:

Equation 1 $RQ=\frac{VCO_{2}}{VO_{2}}=\frac{Ve\cdot FemixCO_{2}}{Vi\cdot FiO_{2}-Ve\cdot FemixO_{2}}$

Combine Equation 5 with Equations 6 and 7:

Equation 5 $Vi=Ve\cdot\frac{FemixN_{2}}{FiN_{2}}=Ve\cdot\frac{1-FemixO_{2}-FemixCO_{2}}{1-FiO_{2}}$

Replace Vi in equation 1 by the expression of equation 5:

$RQ=\frac{VCO_{2}}{VO_{2}}=\frac{Ve\cdot FemixCO_{2}}{Ve\cdot\left( \frac{1-FemixO_{2}-FemixCO_{2}}{1-FiO_{2}} \right)\cdot FiO_{2}-Ve\cdot FemixO_{2}}$

Ve appears in both the numerator and the denominator and can be cancelled. Then multiply both numerator and the denominator by (1–FiO_2_):

$RQ=\frac{VCO_{2}}{VO_{2}}=\frac{\left( 1-FiO_{2} \right)\cdot FemixCO_{2}}{\left( 1-FemixO_{2}-FemixCO_{2} \right)\cdot FiO_{2}-\left( 1-FiO_{2} \right)\cdot FemixO_{2}}$

The denominator may be simplified since the term FiO_2_ FemixO_2_ appears twice with opposite signs and cancels:

$\left( 1-FemixO_{2}-FemixCO_{2} \right)\cdot FiO_{2}-\left( 1-FiO_{2} \right)\cdot FemixO_{2}=$

$$\left( 1-FemixCO_{2} \right)\cdot FiO_{2}-FemixO_{2}$$

Thus, the final expression is given by:

Equation 8 $RQ=\frac{\left( 1-FiO_{2} \right)\cdot FemixCO_{2}}{\left( 1-FemixCO_{2} \right)\cdot FiO_{2}-FemixO_{2}}$

Equations for CO_2_ and O_2_ content in whole blood as described in reference 7

**(The text is taken from reference 7)**

**1.Total carbon dioxide content in whole blood C_t_CO_2 (B)_**

The calculation is based on total CO_2_ in the plasma (P) and erythrocyte fluid (Ery):

C_t_CO_2 (B)_=C_t_C0_2(Ery)_ ^.^ ϕ_Ery(B)_+C_t_C0_2(P)_ ^.^ (1-ϕEry_(B)_)

The volume fraction of erythrocytes i.e., the hematocrit, is estimated from the hemoglobin concentration:

ϕ_Ery(B)_=C_t_Hb_(B)_/C_t_Hb_(Ery)_

C_t_Hb_(Ery)_= 21.00 mmol/l (as per reference 7).

The concentration of total CO_2_ in the erythrocyte fluid is calculated from the erythrocyte pH, the *pCO_2_* and the SO_2_ with a modified Henderson-Hasselbalch equation:

C_t_C0_2(Ery)_=αC0_2(Ery)_ ^.^pCO_2_ ^.^ [1+antilg(pH_Ery_-pK_Ery_)],

pH_Ery_=7.19+0.77 ^.^ (pH_p_-7.4)+0.035 ^.^ (1-SO_2_),

pK_Ery_=6.125-lg [1+anti(pH_Ery_-7.84-0.06 ^.^ SO_2_)],

αC0_2(Ery)_=0.195 mmol l^-1^ kPa^-1^

Equation for pH_Ery_ is a modification of a previously published equation as per reference 7. Equation for pK_Ery_ represents an adaptation to total CO_2_ values calculated with the Singer& Hastings nomogram as per reference 7. The calculation of pH_Ery_ form pHp is based on a normal concentration of 2.3-diphosphoglycerate in the erythrocytes (_CDPG(Ery)_ ≈ 5 mmol/l). A higher DPG concentration gives a higher Donnan potential and greater difference between pH_Ery_ and pH_p_.

**2.Total oxygen content in whole blood CtO_2_**

Calculated as the sum of free and bound oxygen:

c_t_0_2_ = α0_2_ ^.^pO_2_ +C_Hb_  ^.^ SO_2_

C_Hb_  =C_tHb_ ^.^ (1-χ_HbCO_-χ_Hi_)

α0_2_ =0.00983 mmol l^-1^ kPa^-1^

CHb may be termed the concentration of “functional’’ hemoglobin. The most accurate result is obtained if pO_2_ and SO_2_ are both measured.
